# Liposomes as Gene Delivery Vectors for Human Placental Cells

**DOI:** 10.3390/molecules23051085

**Published:** 2018-05-04

**Authors:** Lucie Valero, Khair Alhareth, Jenifer Espinoza Romero, Warren Viricel, Jeanne Leblond, Audrey Chissey, Hélène Dhotel, Caroline Roques, Danielle Campiol Arruda, Virginie Escriou, Nathalie Mignet, Thierry Fournier, Karine Andrieux

**Affiliations:** 1 Chimie ParisTech, PSL Research University, University de Technologies Chimiques et Biologiques pour la Santé (UTCBS), F-75005 Paris, France; lucie.valero@parisdescartes.fr (L.V.); khairallah.alhareth@parisdescartes.fr (K.A.); jenifer.espinoza@etu.univ-lyon1.fr (J.E.R.); Helene.Dhotel@parisdescartes.fr (H.D.); caroline.roques@parisdescartes.fr (C.R.); danielle.campiol-arruda@parisdescartes.fr (D.C.A.); virginie.escriou@parisdescartes.fr (V.E.); nathalie.mignet@parisdescartes.fr (N.M.); 2 CNRS, UTCBS UMR 8258, F-75006 Paris, France; 3 Paris Descartes University, Sorbonne-Paris-Cité, F-75006 Paris, France; 4 INSERM, UTCBS U 1022, F-75006 Paris, France; 5 INSERM, UMR-S1139, Faculty of pharmacy, F-75006 Paris, France; audrey.chissey@parisdescartes.fr (A.C.); Thierry.fournier@parisdescartes.fr (T.F.); 6 Laboratoire de Vectorisation Génétique, Faculty of pharmacy, Montreal University, Montreal, QC H3C 3J7, Canada; viricel.warren@gmail.com (W.V.); jeanne.leblond-chain@umontreal.ca (J.L.); 7 Foundation PremUp, F-75006 Paris, France

**Keywords:** liposomes, siRNA, placenta, primary trophoblast cells

## Abstract

Nanomedicine as a therapeutic approach for pregnancy-related diseases could offer improved treatments for the mother while avoiding side effects for the fetus. In this study, we evaluated the potential of liposomes as carriers for small interfering RNAs to placental cells. Three neutral formulations carrying rhodamine-labelled siRNAs were evaluated on an in vitro model, i.e., human primary villous cytotrophoblasts. siRNA internalization rate from lipoplexes were compared to the one in the presence of the lipofectamine reagent and assessed by confocal microscopy. Results showed cellular internalization of nucleic acid with all three formulations, based on two cationic lipids, either DMAPAP or CSL-3. Moreover, incubation with DMAPAP+AA provided a rate of labelled cells as high as with lipofectamine (53 ± 15% and 44 ± 12%, respectively) while being more biocompatible. The proportion of cells which internalized siRNA were similar when using DMAPAP/DDSTU (16 ± 5%) and CSL-3 (22 ± 5%). This work highlights that liposomes could be a promising approach for gene therapy dedicated to pregnant patients.

## 1. Introduction

Surveys of the world’s clinical trial registries showed pregnant patients are still under-represented during medication development, and particularly in evaluation and safety trials [1,2]. The routine exclusion of pregnant women from clinical trials and the absence of post-marketing surveillance are leading to a huge lack of information regarding drug efficiency and safety for both the fetus and mother [3]. In addition, pregnancy disorders, like preeclampsia, placental insufficiency, viral infection, trophoblastic cancer, and fetal growth restriction, remain without curative treatment and cause considerable health issues [4]. There is, thus, a critical need to encourage research projects aiming at providing additional preclinical safety data and developing new therapeutic strategies. More specifically, drug delivery approaches could carry new active molecules to the placenta, an organ involved in many pregnancy disorders.

Nanomedicine is expected to increase the distribution specificity of therapeutic molecules while reducing their off-target distribution therefore improving therapeutic efficacy and safety [5,6]. The advantages displayed by nanocarriers appear well-suited for pregnancy disorder treatments. Rationally designed nanocarriers could meet the essential requirements: providing an optimized treatment for the mother while avoiding side effects for the fetus by limiting the drug’s transplacental passage [7].

Few reports have been published about the application of nanomedicine for the treatment of pregnancy disorders. We listed and discussed in a recent review the potential of nanomedicine, and particularly liposomes, to empower new strategies for the treatment of preeclampsia, one of the most severe pregnancy complications often responsible for prematurity [7]. We recently reported that PEGylated liposomes penetrate the outer cell layer of the placenta without gaining access to the fetal circulation [8]. This finding support the potential of liposomes as carriers of therapeutic molecules, including gene therapy agents, such as small interfering RNA (siRNA).

RNAi-based medicine holds a pivotal place in the future treatment of human diseases. siRNA are double-stranded RNA oligonucleotides able to induce the silencing of a target gene via RNA interference. Specificity provided by the siRNA sequence could be exploited in the development of therapeutic strategies for pregnancy disorders [9]. However, their instability in biological fluids, as well, as their hydrophilicity, hinder delivery to the target tissue and cellular uptake. Liposome-based formulations could offer the opportunity (i) to protect fragile nucleic acids from nucleases degradation; (ii) to target siRNA delivery to the outer cell layer of placenta villi (the syncytiotrophoblast); and (iii) to restrict off-target effects especially towards the fetus.

In this work we evaluated the cellular internalization of siRNA carried by three different liposome-based formulations. All three formulations are designed to reach neutrality after siRNA loading and have previously been shown to favor transfection with a limited toxicity on other cell types [10,11,12]. Formulations complexed with a fluorescently-labelled siRNA were incubated in vitro with primary human villous cytotrophoblasts. Cells were then observed using confocal microscopy in order to estimate the rate of siRNA internalization into the cells.

## 2. Results

### 2.1. Formulation Characterization

In this study, we selected three formulations previously developed for gene delivery purposes aiming at avoiding the well-known toxicity associated to cationic lipoplexes. The preparation methods and compositions were used as described in [10,11,12], and they are based on the addition of specific lipids and polymers. DMAPAP is a lipopolyamine chosen from a library of cationic lipids and used in our laboratory for its reduced toxicity and high reproducibility as an in vitro transfecting agent [13]. DDSTU is a lipothiourea designed to interact with nucleic acids through hydrogen bond interactions, providing high cargo release efficacy and low toxicity [14]. CSL-3 is a pH-dependent switchable lipid able to complex siRNA and to release it following a pH change [15]. DMAPAP+AA formulation contained 50% of DMAPAP and 5% of PEGylated lipid. An anionic polysaccharide, sodium alginate (AA), was added concurrently to siRNA when preparing DMAPAP+AA, to increase the silencing efficiency and decrease cellular toxicity. DMAPAP/DDSTU formulation was composed of 25% of DMAPAP, 70% of DDSTU, and 5% of PEGylated lipid; mixing DMAPAP and DDSTU is expected to bring both internalization and release abilities. The CSL-3 formulation contained 50% of CLS3 as the cationic lipid and 2.5% of PEGylated lipid. Complete compositions and preparation methods are described in the Materials and Methods (see Section 4.1 and Section 4.2).

Physicochemical characterization was performed with or without siRNA, referred to as lipoplexes and liposomes, respectively. The hydrodynamic diameter, polydispersity index (PdI), zeta potential, and encapsulation rate are shown in Table 1. The hydrodynamic diameters of the DMAPAP-based formulations were around 100 nm, while the diameters of CSL-3-based formulations were around 170 nm. After siRNA complexation, a size increase was observed for all three formulations. Zeta potentials of all formulations were moderately positive and reached neutrality after siRNA addition due to electrostatic interactions between cationic liposomes and siRNA, indicating complexation. Complexation was confirmed and quantified by RiboGreen^®^ assay. The results showed that the siRNA was not available for the fluorescent probe, indicative of a full complexation of siRNA in the three nanoconstructs.

### 2.2. siRNA Delivery to Primary Cytotrophoblasts

The placenta is the interface organ connecting maternal and fetal circulations and controlling the passage of gas, nutrients, and xenobiotics. The outer cell layer, the syncytiotrophoblast (ST), is in direct contact with the mother’s blood flow containing the administered drugs. The culture of human primary villous cytotrophoblasts (VCT) is the most relevant in vitro model in the context of the formulation evaluation for pregnancy-related disease therapy. Indeed, the VCT differentiates in vitro to form a functional syncytiotrophoblast by cell fusion.

**Figure 1:**
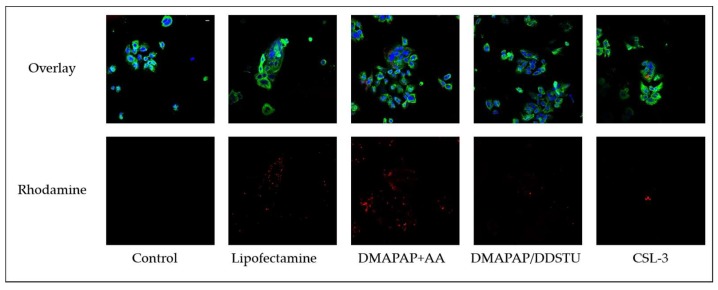
Confocal images comparing siRNA internalization into villous cytotrophoblasts (40×) depending on the formulation (scale bar 10 µm). Nuclei were labelled with DAPI (blue), cytokeratin 7, i.e., cytoskeleton, with Alexa 488 (green), and siRNA with rhodamine (red).

To evaluate the delivery efficiency of the three formulations, villous cytotrophoblasts were incubated with formulations loaded with rhodamine-labelled siRNA (Figure 1). Lipofectamine, a commercial cationic lipid-based reagent, is commonly used for in vitro cells transfection, including villous cytotrophoblasts [16]. Lipofectamine was used in this study as a delivery-positive control. Due to the toxicity of lipofectamine, incubation time must be limited to a maximum of 5 h.

Confocal microscopy observations showed that all tested formulations led to fluorescent siRNA uptake in the primary cytotrophoblasts. The observed rhodamine fluorescence intensity in the cells incubated with the three formulations for 24 h is visually comparable to the one obtained with lipofectamine after 5 h. Therefore, the three tested formulations are able to deliver siRNA to human primary cytotrophoblasts.

**Figure 2:**
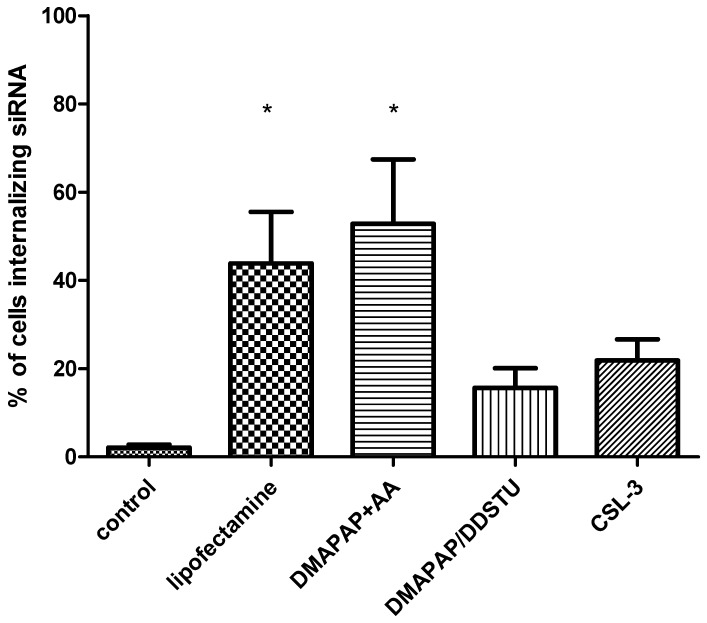
Percentage of observed cells internalizing siRNA. Results are expressed as mean ± standard error from values of experiments realized on cells collected from four placentae. Kruskal-Wallis Dunn’s multiple comparison test: lipofectamine vs. control, *p* < 0.05 (*); DMAPAP+AA vs. control, *p* < 0.05 (*); DMAPAP/DDSTU and CSL-3 vs. control, non-significant.

In order to compare the formulations, a semi-quantitative analysis was performed on confocal images. The percentage of VCT internalizing siRNA was calculated based on the detection of the rhodamine signal into cells marked with cytokeratin 7, a specific marker of human trophoblasts (Figure 2).

Results showed that 44 ± 12% of the VCT were labelled following incubation with siRNA-lipofectamine. Association of siRNA with DMAPAP+AA led to 53 ± 15% of labelled cells, while DMAPAP/DDSTU led to 16 ± 5% and CSL-3 led to 22 ± 5% of labelled cells, respectively. The rate of labelled cells was significantly higher with DMAPAP+AA and with lipofectamine than the control. With DMAPAP/DDSTU and CSL-3, siRNA presented a similar cellular internalization rate.

## 3. Discussion

In this paper, we report for the first time siRNA internalization into human primary cytotrophoblasts using liposomes as nanovectors. The tested formulations were previously developed and designed for in vivo applications. Results showed that DMAPAP+AA, DMAPAP/DDSTU, and CSL-3 had similar or lower delivery efficiency compared to the commercial reagent, lipofectamine. In addition, these formulations are serum compatible allowing an extended incubation time to 24 h in comparison to lipofectamine (5 h maximum). A high tolerance of the lipoplexes formulations was previously reported in vitro with cells from non-placental origin [10,11,12]. The biocompatibility was also confirmed upon intravenous injection on various in vivo mice models, no sign of hemolysis was observed [10,12,17]. Further experiments to study the toxicity of lipoplexes on VCT should be performed.

Various in vitro evaluation models have been set up for the study of placental functions. Many placental cell lines, like BeWo (from choriocarcinoma origin) or HTR8 (immortalized extravillous cytotrophoblasts), can be used, but have a restricted ability to express all necessary functions and/or fail to form a functional and physiological syncytium. To overcome these limitations, the use of human primary villous cytotrophoblasts purified from human placentae seems to be the most suitable to evaluate the potency of different siRNA carriers. This model presents the advantage of both expressing a human phenotype and being representative of morphological and functional differentiation of trophoblastic cells. In addition, villous cytotrophoblasts fuse in vivo to form the syncytiotrophoblast layer which is in direct contact with the maternal circulation. A syncytiotrophoblast is obtained in vitro by the fusion of primary VCT [18]. Moreover, no interspecies data extrapolation is needed thanks to the human origin of this model.

Primary cells are difficult to transfect by non-viral vectors. Fused cells, such as syncytiotrophoblasts with specific barrier properties, were not expected to efficiently allow non-viral vectors to be internalized and deliver their content. Actually, numerous transfection reagents have been tested on placental primary cells and showed highly variable levels of internalization efficacy and reproducibility [16].

In our first study, we showed that liposomes are good candidates as vectors for placental drug delivery thanks to their interaction with the syncytiotrophoblast without the transfer of formulation components to the fetus [8]. In this study, we demonstrate that siRNA loaded into lipoplexes are internalized by VCT. In a previous report, Bajoria et al. studied the internalization of liposomes loaded with a small hydrophilic molecules on human trophoblasts. The authors showed that liposomes were internalized by an energy-dependent pathway and suggested endocytosis as one of the placental cells uptake mechanisms [19]. The internalization pathway for the lipoplexes should be investigated, with endocytosis being highly probable, as this mechanism was previously reported for lipoplexes internalization in other cell types [20].

Moreover, another step in developing these new drug delivery strategies for pregnancy diseases will be to study the efficiency of gene silencing using a therapeutic siRNA. Indeed, nucleic acid internalization is only a part of the complex gene delivery process and does not necessarily imply knock-down of the gene of interest. A potential correlation between cellular internalization and transfection efficiency is however probable with the tested formulations as in vivo gene silencing was demonstrated for two of them in previous investigations [10,12,17].

Further experiments are needed to evidence the absence of placental in-depth penetration and transplacental passage of the siRNA using complementary models, like the human placental explants and the human perfused placenta.

To conclude, our results show that well designed lipoplexes are prone to deliver siRNA into the syncytiotrophoblast, a key target site in many pregnancy-related pathologies. The development of safe and efficient formulations require more investigations and more knowledge in both nanomedicine and pregnancy pathologies.

## 4. Materials and Methods

### 4.1. Materials

Cationic lipids DMAPAP (2-{3-[Bis-(3-amino-propyl)-amino]-propylamino}-*N*-ditetradecyl carbamoyl methyl-acetamide, or dimyristoylaminopropylaminopropyl), and CSL-3 (*N*^1^-(2,6-Bis(5-dodecyl-2-methoxyphenyl)pyridin-4-yl)-*N*^1^,*N*^2^,*N*^2^-trimethylethane-1,2-diamine, or Cationic Switchable Lipid 3) were synthesized as described in previous publications [15,21]. Thiourea lipid DDSTU (2-[2-(didecylamino)-2-oxo-ethoxy]-*N *-[2-(2,3-dihydroxypropylcarbamothioylamino)-1-[(2,3-dihydroxypropylcarbamothioylamino)methyl]ethyl]acetamide, or didecylserinethiourea) was synthesized as described in the work of Breton et al. [14]. PEGylated lipid Dodagly-PEG ((2-dioctadecylcarbamoyl-methoxyacetylamino) acetic acid-(α-methoxy)-polyethylene glycol 2000 ester) was synthesized according to previous protocol [22]. Commercial lipids DOPE (1,2-dioleoyl-sn-glycero-3-phosphoethanolamine), DSPC (1,2-distearoyl-sn-glycero-3-phosphocholine) and cholesterol were purchased from Avanti Polar Lipids (Alabaster, AL, USA). Dulbecco’s modified eagle’s and optiMEM medium, Hank’s balanced salt solution (HBSS), glutamine, penicillin streptomycin, Triton X100, and sodium alginate were purchased from Sigma Aldrich (Saint Quentin Fallavier, France). Primary and secondary antibodies, and DAPI, were obtained from Life Technologies (Saint Aubin, France). Paraformaldehyde (PFA) was acquired from Electron Microscopy Sciences (Hatfield, UK). Fetal calf serum (FCS) was purchased from Eurobio (Courtaboeuf, France).

### 4.2. Liposome and Lipoplex Preparation

Three liposome formulations were prepared: DMAPAP+AA, DMAPAP/DDSTU and CLS3-50 according to previous reports [10,11,12]. Briefly, for DMAPAP+AA, lipids (DMAPAP/DOPE/Dodagly-PEG 50/45/5, molar ratio) were dissolved in chloroform and the organic solvent was evaporated to form a thin film. The dried film was hydrated with 150 mM NaCl and extruded to obtain a homogenous suspension of liposomes (2.5 mM). For the DMAPAP/DDSTU formulation, liposomes were prepared by the ethanolic injection method with the specific molar ratio: DMAPAP/ DDSTU/Dodagly-PEG, 25/70/5, in order to obtain a final lipid concentration of 2.5 mM in 150 mM NaCl. For the CLS3-50 formulation, liposomes were prepared using the thin film method with a lipid composition and molar ratio of CSL3/DSPC/cholesterol/Dodagly-PEG 50/10/37.5/2.5. The dried film was hydrated with 5% dextrose, sonicated and extruded, and the final concentration was adjusted to 2.5 mM. Formulation preparation methods and/or hydration buffer differ from one-another as we intended to follow precisely the protocols described in [10,11,12].

Lipoplexes were prepared by mixing an equal volume of the siRNA solution (plus sodium alginate for the DMAPAP+AA formulation) to the cationic liposome suspension and rapidly mixed by vortexing. When using lipofectamine 2000, the same steps were followed, and the diluted transfection reagent was mixed with an equal volume of siRNA solution. According to the manufacturer’s instructions, ratio was 1 µL of lipofectamine for 5 pmol of siRNA. Lipoplexes were incubated for 30 min at room temperature for the DMAPAP+AA and DMAPAP/DDSTU formulations and lipofectamine, 10 min at 50 °C for the CSL-3 formulation. The charge ratio was N/P = 4 for DMAPAP+AA, DMAPAP/DDSTU, and CSL-3, and was calculated based on the molar ratio of positive charges (three positive charges per molecule of DMAPAP, one per molecule of CSL3) and the molar ratio of negative charges (from siRNA and sodium alginate molecules, 3.08 nmol negative charges per µg and 5.05 nmol negative charges per µg, respectively).

### 4.3. Liposome and Lipoplex Characterization

Mean particle hydrodynamic diameter (Z-average), polydispersity index (PdI), and zeta potential were determined using a Malvern Zetasizer Nano ZS (Malvern Instruments Ltd., Malvern, UK). Before each measurement, an appropriate dilution (1/100) of liposomes or lipoplexes suspensions was performed in NaCl 150 mM. The encapsulation efficiency of siRNA in the formulations was determined by RiboGreen^®^ assay according to the manufacturer’s instructions (Invitrogen, Carlsbad, CA, USA).

### 4.4. Placentae Collection

Placentae were collected from women with normal pregnancies, following caesarean section (between 37 and 41 weeks of gestation). Biological samples were obtained after informed patient written consent and approval from our local ethics committee (CCP-2015-Mai-13909).

### 4.5. Primary Villous Cytotrophoblasts Isolation and Purification

Primary villous cytotrophoblasts (VCT) isolation by differential trypsin digestion was performed as previously described [23]. Chorionic villi were washed, dissected, and rinsed in Ca^2+^, Mg^2+^-free HBSS, then incubated sequentially with trypsin. Each time, the supernatant containing VCT was collected after tissue sedimentation, filtered (100 μm), and incubated with 10% FCS (*v*/*v*) to stop trypsin activity. After Percoll gradient fractionation, cells were diluted to a concentration of 1 × 10^6^ cells/mL in DMEM supplemented with 10% FCS, 2 mM glutamine, 100 UI/mL penicillin, and 100 μg/mL streptomycin. Cells were then plated at a density of 150,000 cells/cm^2^ on 60 mm culture dishes. VCT were incubated overnight in 5% CO_2_ at 37 °C and washed three times with PBS to eliminate non-adherent cells. Culture purity was assessed by immunolabelling with anti-CK7 antibody (a trophoblast-specific marker). The quality of the culture and formation of the ST was monitored by hCG quantification in cell supernatants.

### 4.6. In Vitro siRNA Internalization

VCT were incubated with rhodamine-labelled nonspecific siRNA molecules (CGGCAAGCTGACCCTGAAGTTCAT) (Qiagen, Courtaboeuf, France) at a final siRNA concentration of 20 nM. Lipofectamine 2000 transfection reagent was used according to the manufacturer’s instructions, i.e., using optiMEM medium without FCS, and the incubation time was 5 h. For the three prepared formulations, an adequate concentration of lipoplexes suspension was diluted into optiMEM with FCS to reach the siRNA final concentration. Incubation with the formulation lasted 24 h. Experiments were performed on cells isolated from *n* = 4 placentae. After 5 h of cell incubation, an observation by fluorescence microscope was performed showing a fluorescence signal for all liposomal and lipofectamine formulations of siRNA (data not shown).

### 4.7. Confocal Microscopy

After 48 h of primary culture, VCT were fixed in 4% paraformaldehyde for 20 min, then paraformaldehyde was neutralized with NH_4_Cl 50 mM for 10 min. Cells were rinsed three times with PBS and permeabilized in Triton 0.1% for 10 min. Saturation of unspecific sites was performed in PBS with 1% BSA and 0.1% Tween at room temperature. Primary antibodies against cytokeratin 7 were added for 2 h at room temperature and amplified with the Alexa Fluor 488^®^-conjugated secondary antibody for 2 h at room temperature. Nuclei were then stained with DAPI. Cells were mounted in a fluorescent Dako mounting medium (Dako, Glostrup, Denmark), examined, and photographed on a TCS SP2 confocal microscope (Leica, Wetzlar, Germany) of the Cellular and Molecular Imaging facility, INSERM UMS 025—CNRS UMS 3612, Faculté de Pharmacie de Paris, Université Paris Descartes, Paris, France.

Semi-quantitative assessment was performed counting at least 100 cells per condition per placentae. Statistical tests were performed using GraphPad Prism (GraphPad Software Inc. 5.01, La Jolla, CA, USA).

## Figures and Tables

**Table 1:** Characterization of the liposomes and lipoplexes formulations.

	DMAPAP+AA		DMAPAP/DDSTU		CSL-3	
	Liposomes	Lipoplexes	Liposomes	Lipoplexes	Liposomes	Lipoplexes
Mean hydrodynamic diameter (nm)	104 ± 4	121 ± 2	72 ± 4	86 ± 7	170 ± 9	317 ± 7
Polydispersity Index	0.25 ± 0.02	0.18 ± 0.01	0.22 ± 0.01	0.24 ± 0.02	0.25 ± 0.01	0.25 ± 0.01
Zeta potential (mV)	28 ± 4	−5 ± 2	12 ± 5	−2 ± 0.4	31 ± 2	−2 ± 1
siRNA encapsulation (%)		100		100		98

## Abbreviations:

DMAPAP	dimyristoylaminopropylaminopropyl
CSL-3	cationic switchable lipid 3
DDSTU	didecylserinethiourea
VCT	primary villous cytotrophoblasts

## References

[1] Shields K.E., Lyerly A.D. (2013). Exclusion of pregnant women from industry-sponsored clinical trials. Obstet. Gynecol..

[2] Scaffidi J., Mol B., Keelan J. (2017). The pregnant women as a drug orphan: A global survey of registered clinical trials of pharmacological interventions in pregnancy. BJOG Int. J. Obstet. Gynaecol..

[3] Baylis F., MacQuarrie R. (2016). Why Physicians and Women Should Want Pregnant Women Included in Clinical Trials.

[4] Keelan J.A., Leong J.W., Ho D., Iyer K.S. (2015). Therapeutic and safety considerations of nanoparticle-mediated drug delivery in pregnancy. Nanomedicine.

[5] Lamichhane N., Udayakumar T., D’Souza W., Simone C., Raghavan S., Polf J., Mahmood J. (2018). Liposomes: Clinical Applications and Potential for Image-Guided Drug Delivery. Molecules.

[6] Alhareth K., Sancey L., Tsapis N., Mignet N. (2017). How should we plan the future of nanomedicine for cancer diagnosis and therapy?. Int. J. Pharm..

[7] Valero L., Alhareth K., Gil S., Lecarpentier E., Tsatsaris V., Mignet N., Fournier T., Andrieux K. (2018). Nanomedicine as a potential approach to empower the new strategies for the treatment of preeclampsia. Drug Discov. Today.

[8] Valero L., Alhareth K., Gil S., Simasotchi C., Roques C., Scherman D., Mignet N., Fournier T., Andrieux K. (2017). Assessment of dually labelled PEGylated liposomes transplacental passage and placental penetration using a combination of two *ex-vivo* human models: The dually perfused placenta and the suspended villous explants. Int. J. Pharm..

[9] Yu J., Jia J., Guo X., Chen R., Feng L. (2017). Modulating circulating sFlt1 in an animal model of preeclampsia using PAMAM nanoparticles for siRNA delivery. Placenta.

[10] Schlegel A., Largeau C., Bigey P., Bessodes M., Lebozec K., Scherman D., Escriou V. (2011). Anionic polymers for decreased toxicity and enhanced in vivo delivery of siRNA complexed with cationic liposomes. J. Controll. Release.

[11] Seguin J., Dhotel H., Kai-Luen R., Bessodes M., Mignet N. (2015). Fine tuning of mixed ionic and hydrogen bond interactions for plasmid delivery using lipoplexes. Eur. J. Pharm. Biopharm..

[12] Viricel W., Poirier S., Mbarek A., Derbali R.M., Mayer G., Leblond J. (2017). Cationic switchable lipids: PH-triggered molecular switch for siRNA delivery. Nanoscale.

[13] Byk G., Dubertret C., Escriou V., Frederic M., Jaslin G., Rangara R., Pitard B., Crouzet J., Wils P., Schwartz B. (1998). Synthesis, Activity, and Structure−Activity Relationship Studies of Novel Cationic Lipids for DNA Transfer. J. Med. Chem..

[14] Breton M., Leblond J., Seguin J., Midoux P., Scherman D., Herscovici J., Pichon C., Mignet N. (2010). Comparative gene transfer between cationic and thiourea lipoplexes. J. Gene Med..

[15] Viricel W., Mbarek A., Leblond J. (2015). Switchable Lipids: Conformational Change for Fast pH-Triggered Cytoplasmic Delivery. Angew. Chem. Int. Ed..

[16] Forbes K., Desforges M., Garside R., Aplin J.D., Westwood M. (2009). Methods for siRNA-mediated Reduction of mRNA and Protein Expression in Human Placental Explants, Isolated Primary Cells and Cell Lines. Placenta.

[17] Bedarida T., Domingues A., Baron S., Ferreira C., Vibert F., Cottart C.-H., Paul J.-L., Escriou V., Bigey P., Gaussem P. (2018). Reduced endothelial thioredoxin-interacting protein protects arteries from damage induced by metabolic stress in vivo. FASEB J..

[18] Orendi K., Kivity V., Sammar M., Grimpel Y., Gonen R., Meiri H., Lubzens E., Huppertz B. (2011). Placental and trophoblastic in vitro models to study preventive and therapeutic agents for preeclampsia. Placenta.

[19] Bajoria R., Sooranna S.R., Contractor S.F. (1997). Endocytotic uptake of small unilamellar liposomes by human trophoblast cells in culture. Hum. Reprod..

[20] Pinnapireddy S.R., Duse L., Strehlow B., Schäfer J., Bakowsky U. (2017). Composite liposome-PEI/nucleic acid lipopolyplexes for safe and efficient gene delivery and gene knockdown. Colloids Surf. B Biointerfaces.

[21] Mignet N., Richard C., Seguin J., Largeau C., Bessodes M., Scherman D. (2008). Anionic pH-sensitive pegylated lipoplexes to deliver DNA to tumors. Int. J. Pharm..

[22] Mignet N., Seguin J., Ramos Romano M., Brullé L., Touil Y.S., Scherman D., Bessodes M., Chabot G.G. (2012). Development of a liposomal formulation of the natural flavonoid fisetin. Int. J. Pharm..

[23] Handschuh K., Guibourdenche J., Tsatsaris V., Guesnon M., Laurendeau I., Evain-Brion D., Fournier T. (2007). Human Chorionic Gonadotropin Produced by the Invasive Trophoblast But Not the Villous Trophoblast Promotes Cell Invasion and Is Down-Regulated by Peroxisome Proliferator-Activated Receptor-γ. Endocrinology.

